# Investigation of a Novel *LRP6* Variant Causing Autosomal-Dominant Tooth Agenesis

**DOI:** 10.3389/fgene.2021.688241

**Published:** 2021-07-07

**Authors:** Yan-xia Huang, Chun-yan Gao, Chun-yan Zheng, Xu Chen, You-sheng Yan, Yong-qing Sun, Xing-yue Dong, Kai Yang, Dong-liang Zhang

**Affiliations:** ^1^Department of Orthodontics, School of Stomatology, Beijing Stomatological Hospital, Capital Medical University, Beijing, China; ^2^Prenatal Diagnosis Center, Beijing Obstetrics and Gynecology Hospital, Capital Medical University, Beijing, China

**Keywords:** tooth agenesis, *LRP6* gene, whole-exome sequencing, molecular dynamics analysis, experimental study

## Abstract

**Background:**

The low-density lipoprotein receptor-related protein 6 (*LRP6*) gene is a recently defined gene that is associated with the autosomal-dominant inherited tooth agenesis (TA). In the present study, a family of four generations having TA was recruited and subjected to a series of clinical, genetic, *in silico*, and *in vitro* investigations.

**Methods:**

After routine clinical evaluation, the proband was subjected to whole-exome sequencing (WES) to detect the diagnostic variant. Next, *in silico* structural and molecular dynamics (MD) analysis was conducted on the identified novel missense variant for predicting its intramolecular impact. Subsequently, an *in vitro* study was performed to further explore the effect of this variant on protein maturation and phosphorylation.

**Results:**

WES identified a novel variant, designated as *LRP6*: c.2570G > A (p.R857H), harbored by six members of the concerned family, four of whom exhibited varied TA symptoms. The *in silico* analysis suggested that this novel variant could probably damage the Wnt bonding function of the LRP6 protein. The experimental study demonstrated that although this novel variant did not affect the *LRP6* gene transcription, it caused a impairment in the maturation and phosphorylation of LRP6 protein, suggesting the possibility of the disruption of the Wnt signaling.

**Conclusion:**

The present study expanded the mutation spectrum of human TA in the *LRP6* gene. The findings of the present study are insightful and conducive to understanding the functional significance of specific *LRP6* variants.

## Introduction

Tooth agenesis (TA) is one of the most prevalent congenital craniofacial malformations occurring in humans, which may lead to masticatory dysfunction, speech alteration, and malocclusion, besides aesthetic problems (Matalova et al., 2008). TA occurs in over 300 syndromic or non-syndromic conditions, with a remarkable phenotypic heterogeneity^1^. Among the familial cases of TA, autosomal dominant inheritance is the most frequent pattern observed (Matalova et al., 2008). On the basis of the number of missing teeth, TA is classified into hypodontia (<6 teeth) and oligodontia (≥6 teeth). Hypodontia is quite common, with a prevalence of 3–10% depending on the population, while oligodontia is rare, with a prevalence of <1% (Dhamo et al., 2018).

Genetic variations contribute greatly to the pathogenesis of congenital TA, and may also provide insights into tooth development (Yin and Bian, 2015). To date, 15 genes in the WNT/β-catenin, TGF-β/BMP, and Eda/Edar/NF-κB pathways, namely, *WNT10A* (MIM ^∗^606268), *WNT10B* (MIM ^∗^601906), *LRP6* (MIM ^∗^603507), *DKK1* (MIM ^∗^605189), *KREMEN1* (MIM ^∗^609898), *AXIN2* (MIM ^∗^604025), *PAX9* (MIM ^∗^167416), *MSX1* (MIM ^∗^142983), *GREM2* (MIM ^∗^608832), *BMP4* (MIM ^∗^112262), *LTBP3* (MIM ^∗^602090), *EDA* (MIM ^∗^300451), *EDAR* (MIM ^∗^604095), *EDARADD* (MIM ^∗^606603), and *SMOC2* (MIM ^∗^607223), have been reported to be responsible for non-syndromic TA (see text footnote 1).

The WNT/β-catenin pathway plays a pivotal role in cell differentiation, proliferation, and migration involved in the formation and homeostasis of bone and teeth (Duan and Bonewald, 2016). The *LRP6* gene encodes the low-density lipoprotein receptor-related protein 6 (LRP6), which is a single-pass transmembrane receptor of Wnts in the WNT/β-catenin pathway (Tamal et al., 2000; Kokubu et al., 2004). The mutations in the *LRP6* gene were initially reported to be associated with a broad spectrum of anomalies in human and animals, such as neural tube defects (Carter et al., 2005), early coronary disease (Mani et al., 2007), and metabolic syndromes (Singh et al., 2013). Massink et al. (2015) reported that the variations in the *LRP6* gene could cause autosomal dominant TA. Later, three studies on TA identified other variants of *LRP6*, which corroborated this causality (Ockeloen et al., 2016; Dinckan et al., 2018; Yu et al., 2020). In the most recent study, Yu et al. (2020) revealed the spatial/temporal expression pattern of Lrp6 in mouse molar development, which provided further insight into the dynamic function of the WNT/β-catenin pathway in tooth formation.

In the present study, a Chinese family with four generations having autosomal dominant TA was recruited and subjected to a comprehensive genetic investigation which revealed a novel missense variant in *LRP6*. In order to confirm the pathogenicity of the identified novel variant and understand its impact on the protein structure and function, western blotting (WB) and *in silico* molecular dynamics (MD) simulation analyses were performed.

## Materials and Methods

### Subjects and Clinical Evaluation

The present study was designed as a prospective review and was approved by the Ethics Committee of the Capital Medical University Affiliated Beijing Stomatological Hospital. Informed consent was provided by all the participants included in the study. All procedures performed in the present study were in accordance with the Declaration of Helsinki 1964 and its later amendments or comparable ethical standards.

A family with four generations having hereditary TA was recruited at the Beijing Stomatological Hospital in December 2019. Twelve members of this family, including seven females and five males, participated in the study. The medical histories of all participants were thoroughly surveyed, and a pedigree diagram was drawn. Panoramic radiography was conducted for the members affected by TA.

### Genetic Studies

Genomic DNA was extracted from the peripheral blood sample from each of the 12 subjects using QIAamp DNA Blood Mini kit (Qiagen, Germany).

Whole-exome sequencing was employed to detect the sequence variants in the sample from the proband, as described in a previous study (Yang et al., 2019). Briefly, the target-region sequences were enriched using the Agilent Sure Select Human Exon Sequence Capture Kit, V5 + UTR (Agilent, United States). The DNA libraries were screened using quantitative PCR, the size, distribution, and concentration of which were determined using Agilent Bioanalyzer 2100 (Agilent, United States). The NovaSeq6000 platform (Illumina, Inc.) and ∼150 bp pair-end reads were employed to sequence the DNA (∼300 pM per sample) using the NovaSeq Reagent kit. The sequencing raw reads (quality level Q30 > 90%)^2^ were aligned to the human reference genome (accession no: hg19/GRCh37) in Burrows–Wheeler Aligner tool, and the PCR duplicates were removed using Picard v1.57. Variant calling was performed using the Verita Trekker^®^ Variants Detection system (v2.0; Berry Genomics, China) and the Genome Analysis Tool Kit^3^ (detailed variant filtering criteria included in Supplementary Material). Subsequently, the annotation tools, ANNOVAR v2.0 (Wang et al., 2010) and Enliven^®^ Variants Annotation Interpretation systems (Berry Genomics), were used to provide information for the establishment of the criteria of the common guidelines provided by the American College of Medical Genetics and Genomics (ACMG) (Richards et al., 2015). In order to assist in the interpretation of pathogenicity, three frequency databases (1000G_2015aug_eas, ExAC_EAS, and gnomAD_exome_EAS)^4^^5^^6^ and HGMD pro v2019 (Human Gene Mutation Database) were referred to. The Revel score (for pathogenicity prediction) (Ioannidis et al., 2016) and the pLI score (representing the tolerance for truncating variants) were also utilized.

As a confirmatory method, Sanger sequencing was performed using the 3500DX Genetic Analyzer (Applied Biosystems, United States). The details regarding the sequencing PCR primers, reaction conditions, and reagents are provided in Supplementary Material. The evolutionary conservatism of the amino acid (AA) residue affected by the identified novel missense variant was analyzed using MEGA7^7^ with the default parameters.

### Structural and Molecular Dynamics (MD) Analysis

The SWISS-MODEL program was applied for modeling the LRP6 PE3/4 domains containing the mutation site, using the crystal structure PDB:3S2K^8^ with X-resolution of 2.8 Å as the template (Ahn et al., 2011).

Next, the molecular dynamics (MD) prediction analysis was performed for the wild type (WT) LRP6 and R857H-LRP6 models generated by Modeler 9v17 (Šali et al., 1995). The CHARMM22 program was applied to add hydrogen atoms and N-terminal and C-terminal patches to the models (MacKerell et al., 1998). The generated models were solvated and neutralized with TIP3P water within a box at a minimum distance of 13 Å between the model and the wall of the box. All simulations were run using NAMD 2.9 and by applying periodic boundary conditions (PBC) (Phillips et al., 2020). The temperature was set at 300 K, the pressure was set at 1 atm, and the time step was set to 2 fs. The particle mesh Ewald method was used for modeling the electrostatics, and the threshold for van der Waals interactions was set at 12 Å. Both the models followed a three-step pre-equilibration totaling 600 ps, with the last snapshots selected as the beginning structures for 60-ns productive simulations without constraints.

### Plasmids Construction, Cell Transfection, and Wnt3a Treatment

In order to construct the expression plasmid vectors containing the coding sequence of the wild-type (WT) or mutant *LRP6*, the cDNA sequences of both WT and mutant *LRP6* were obtained using RT-PCR from the mRNA sample extracted from the proband. The obtained cDNA sequences were subcloned into the pET vectors and verified using Sanger sequencing. Next, the cDNA sequences were inserted into the pcDNA3.1(+) vectors, and the resultants were designated as LRP6-WT and LRP6-Mut, which represented the WT and mutant ones, respectively.

Commercial HEK (human embryonic kidney) 293T cells were purchased and cultured in 24-well plates. Subsequently, the cells were transfected with LRP6-WT or LRP6-Mut using Lipofectamine 3000 (Invitrogen, United States) at a suitable confluence.

This process lasted for 48 h, after which the cells were (**±**) treated with recombinant human Wnt3a protein (Cat No. ab153563, Abcam, United States) via Lipofectamine 3000.

### Western Blotting (WB)

In order to analyze the expression levels and the phosphorylation levels of the LRP6 protein in the HEK 293T cells, WB was performed using the monoclonal antibodies against LRP6 (Cat No. 2560, CST, United States) and phospho-LRP6 (Ser1490) (Cat No. 2568, CST, United States), respectively. Detailed methods were described in Supplementary Material.

### RNA Expression Analysis With Quantitative Fluorescent RT-PCR

At 48 h after transfection, cells were harvested and total RNA was extracted with an RNeasy Mini Kit (QIAGEN). Reverse transcription was performed with the Prime Script RT Reagent Kit with the gDNA Eraser (Takara). The expression level of *LRP6* was assessed by quantitative fluorescent RT-PCR using SYBR Premix Ex Taq II (Perfect Real Time) (Takara) with ABI 7500 system (see details in Supplementary Material).

### Statistical Analysis

Statistical calculations were performed using the SPSS v22.0 software. Student’s *t-*test was used for determining the statistical significance and *p* ≤ 0.05 was considered significant.

## Results

### Clinical Manifestations

**FIGURE 1:**
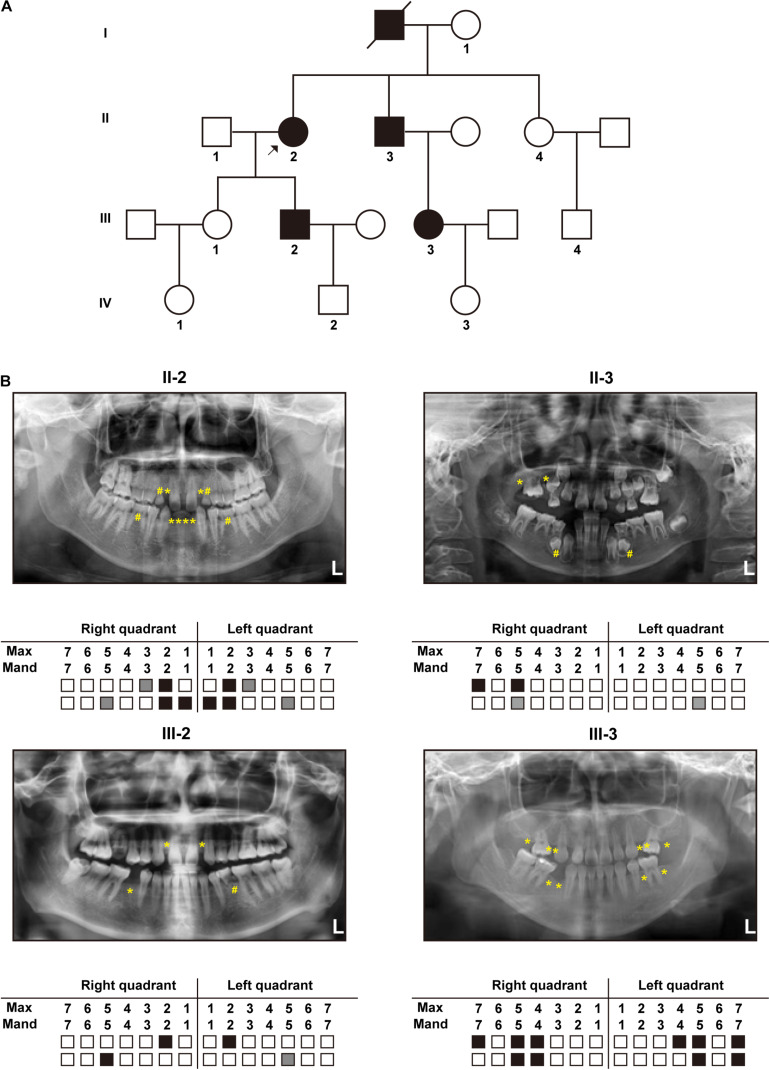
Clinical manifestations: **(A)** Pedigree diagram of the recruited family with autosomal dominant tooth agenesis (TA) (The arrow indicates the proband). **(B)** Panoramic radiographs and schematics of the four patients in the recruited family (White blocks represent the normal eruption of permanent teeth; Gray blocks and “#” marks represent residual deciduous teeth; Black blocks and “*” marks represent permanent tooth agenesis).

The constructed pedigree diagram is depicted in Figure 1A. Four members of the recruited family, designated as II-2 (the proband), II-3, III-2, and III-3, exhibited apparent permanent tooth loss, with a remarkable phenotypic heterogeneity in the number of missing teeth. In addition, both II-2 and III-3 had sparse hair (data not presented as requested by the participants). The phenotype of generation IV was not conclusive as all three subjects in this generation (IV-1, IV-2, and IV-3) were at the age of deciduous teeth. Detailed clinical information regarding the four affected members is provided in Table 1 and the panoramic radiographs and the corresponding schematics are presented in Figure 1B. The images of the four patients in this study were included in Supplementary Material.

**TABLE 1:** Clinical features of the four patients in this family.

Subject ID	Gender	Age	Number of a genetic teeth (excluding third molar)	Other manifestation
II-2	F	61 yr	10	Sparse hair
II-3	M	58 yr	4	NA
III-2	M	29 yr	4	NA
III-3	F	28 yr	10	Sparse hair

*F, female; M, Male; yr, years old; NA, not applicable.*

### Genetic Findings

**FIGURE 2:**
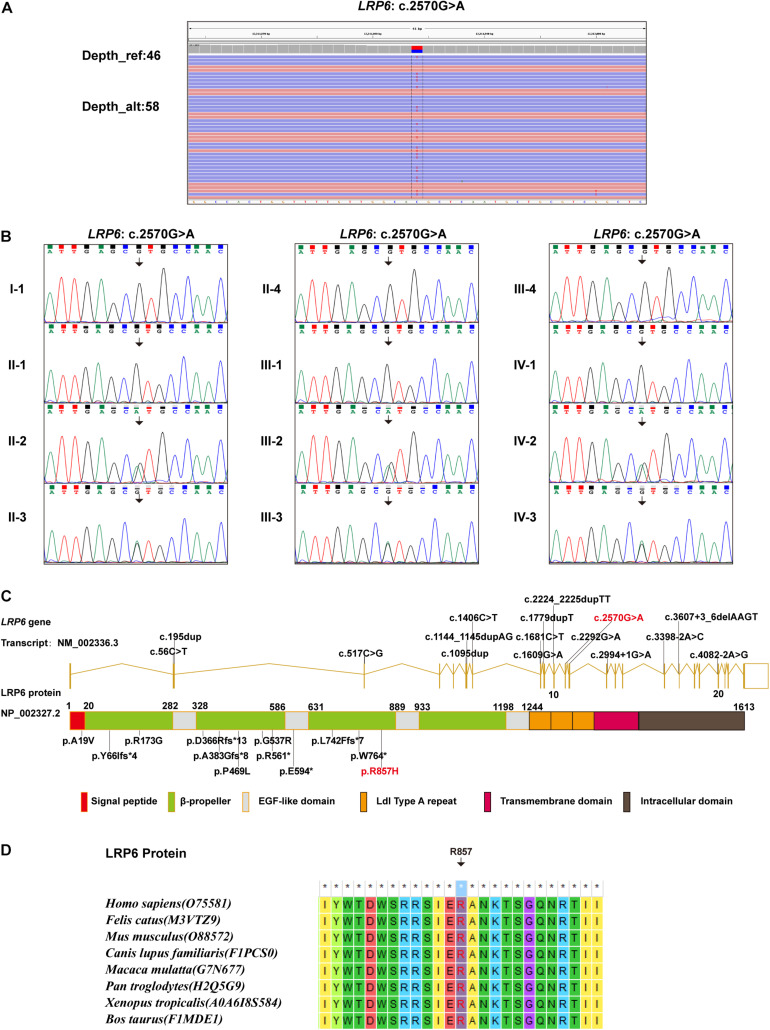
Genetic findings: **(A)** WES data demonstrating the *LRP6*: c.2570G > A variant. **(B)** Sanger sequencing results demonstrating the carrying status of the *LRP6*: c.2570G > A variant in the 12 members of the recruited family. **(C)** All *LRP6* variants associated with TA phenotype reported in literature, illustrated in gene and protein schematics (Red fonts represent the variant in the present study). **(D)** The conservatism of the amino acid (R857) affected by c.2570G > A variant across species.

Whole-exome sequencing identified a novel heterozygous missense variant, which was named *LRP6*: NM: 002336.3: c.2570G > A (p.R857H). This identified variant was later verified using Sanger sequencing (Figures 2A,B). In addition, Sanger sequencing revealed that six members in this family, namely, II-2, II-3, III-2, III-3, IV-2, and IV-3, carried the identified novel variant, while the other six members carried the wild-type variant (Figure 2B). As depicted in the linear structural patterns of the genes and proteins, the mutation was located in the PE3 (P, the YWTD β-propeller domain; E, the epidermal growth factor-like domain) domain (Figure 2C). The AA residue that was affected by this novel mutant variant, namely R857, remained evolutionarily conserved among species (Figure 2D). None of the three frequency databases searched had this variant included, and both REVEL and pLI predictions indicated it as being “deleterious” to gene function (see Supplementary Material). Accordingly, this variant fulfills the evidence level PM2, PP1, PP2, PP3, and PP4.

### The *LRP6*^*R*857*H*^ Variant Impact the Protein Stability and Secondary Structure

**FIGURE 3:**
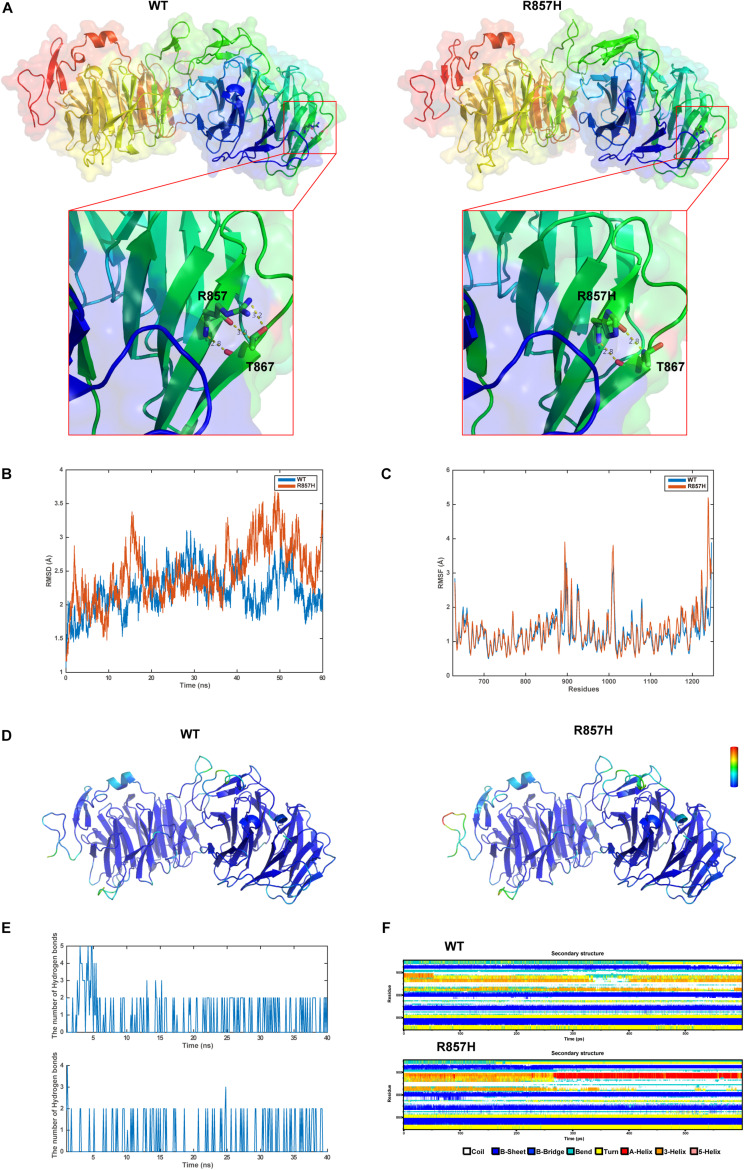
Structural and MD analysis results: **(A)** The structures of the domain containing the WT and R857H models (Residues forming hydrogen bonds with the residue R857 or R857H are depicted in stick representation; Dotted yellow lines represent the hydrogen bonds). **(B)** The trajectory of RMSD (Cα) for the two proteins, which compared every structure in the trajectory to the reference/initial frame (0 ns) by computing the root mean square deviation (RMSD). RMSD is a numerical measurement representing the difference between two structures. In molecular dynamics, we are interested in how structures and parts of structures change over time as compared to the starting point, so the trajectory of RMSD can be used to identify large changes in protein structure as compared to the starting point. **(C)** RMSF of the two proteins calculated from each simulation, which computed the root mean square fluctuation (RMSF) of atomic positions in the trajectory after fitting to the reference/initial frame (0 ns). RMSF is a numerical measurement similar to RMSD, but instead of indicating positional differences between entire structures over time, RMSF is a calculation of individual residue flexibility, or how much a particular residue moves (fluctuates) during a simulation. **(D)** The two models (WT and R857H) colored according to RMSF. **(E)** The number of hydrogen bonds formed between the residue R857 (up) or R857H (blew) and the other residues for each structure in the trajectory. Although the hydrogen bond is much weaker than a covalent bond, the large number of imide and carbonyl groups in peptide chains results in the formation of numerous hydrogen bonds, and these are important for structures to stabilize the folding of the peptide backbone and facilitate molecular interactions. **(F)** Secondary structural components of the corresponding region as a function of time. Secondary structures, refer to local folded structures that form within a polypeptide due to interactions between atoms of the backbone, linked topologically to form 3D structures. The secondary structures are changed after mutation, especially for residues 890–900.

The final and converged models out of structure prediction are depicted in Figure 3A. It could be inferred from the trajectory of Root Mean Square Deviation (RMSD) and Root Mean Square Fluctuation (RMSF) that the R857H model was extremely flexible compared to the WT model (Figures 3B–D). Unsurprisingly, amino acid residue R857 formed a greater number of hydrogen bonds with the other residues compared to the variant residue R857H (Figure 3E). Furthermore, R857H variant affected the corresponding secondary structures of the LRP6 protein (Figure 3F).

### *LRP6*^*R*857*H*^ Impacts the Protein Maturation and Phosphorylation

**FIGURE 4:**
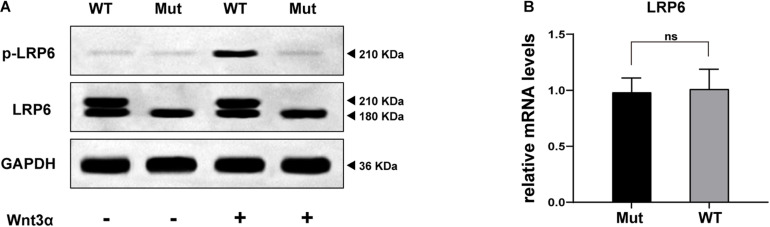
*In vitro* study results: **(A)** WB bands representing the expression, maturation, and phosphorylation levels of LRP6 protein, compared between the two groups of cells (transfected by WT or Mutant LRP6 cDNA, respectively). **(B)** The result of real-time fluorescence quantitative PCR demonstrating the transcriptional level of *LRP6*, compared between the two groups of cells.

As depicted in Figure 4A, both WT and Mut LRP6 vectors could be expressed exogenously, although the LRP6-Mut vector generated only a single lower molecular weight (MW) protein band while the LRP6-WT vector generated two adjacent bands. Moreover, after treatment with Wnt3a, a significantly higher amount of the phosphorylated LRP6 protein was produced by WT compared to the Mut.

Figure 4B presents the comparison of the transcription levels of *LRP6*, with no significant difference indicated.

## Discussion

The *LRP6* gene has been cloned and studied for several decades now (Brown et al., 1998; Pinson et al., 2000). Nonetheless, the comprehensive function of this gene in the physiological process of humans remains to be elucidated. The possibility of the pathogenic variants in *LRP6* leading to autosomal dominant TA was revealed quite recently (Massink et al., 2015). So far, to the best of our knowledge, only 15 TA-related pathogenic variants in *LRP6* are reported, which includes seven truncating variants, four missense variants, and four splicing variants (Figure 2C and Table 2; Massink et al., 2015; Ockeloen et al., 2016; Dinckan et al., 2018; Yu et al., 2020). The present study is the fifth one on TA that reports the identification of a novel *LRP6* variant. Most mutations (11 of 16) are loss of function mutations (frameshift or splice site), although a dominant negative effect of mutations has been suggested by other authors (Yu et al., 2020). Despite the current number of variants and their distribution on various domains, and the evidences provided by functional experiments, the genotype-phenotype correlations of LRP6 gene still cannot be well established.

**TABLE 2:** Tooth agenesis-related variants in the *LRP6* gene (cited from HGMD database, in chronological order of reports).

No.	Genomic coordinates	Reference base (s)	Variant base (s)	HGVS description (NM_002336.3)	Protein alteration	References (PMID)
1	12:12315180-12315180	C	CAA	c.2224_2225dupTT	p.L742Ffs*7	PMID: 26387593
2	12:12397589-12397589	G	A	c.56C > T	p.A19V	PMID: 26387593
3	12:12317479-12317479	C	CA	c.1779dupT	p.E594*	PMID: 26387593
4	12:12334204-12334204	C	CCT	c.1144_1145dupAG	p.A383Gfs*8	PMID: 26387593
5	12:12318166-12318166	C	T	c.1609G > A	p.G537R	PMID: 26963285
6	12:12291470-12291470	T	G	c.3398-2A > C	Splice site	PMID: 26963285
7	12:12303769-12303769	C	T	c.2994 + 1G > A	Splice site	PMID: 26963285
8	12:12356267-12356267	G	C	c.517C > G	p.R173G	PMID: 26963285
9	12:12332883-12332883	G	A	c.1406C > T	p.P469L	PMID: 26963285
10	12:12279857-12279857	T	C	c.4082-2A > G	Splice site	PMID: 26963285
11	12:12291252-12291256	CACTT	C	c.3607 + 3_6delAAGT	Splice site	PMID: 28813618
12	12:12312886-12312886	C	T	c.2292G > A	p.W764*	PMID: 33164649
13	12:12397449-12397450	T	TT	c.195dup	p.Y66Ifs*4	PMID: 33164649
14	12:12334254_12334255	T	TT	c.1095dup	p.D366Rfs*13	PMID: 33164649
15	12:12318094-12318094	G	A	c.1681C > T	p.R561*	PMID: 33164649
16	12:12311984-12311984	C	T	c.2570G > A	p.R857H	This study

*HGMD, The Human Gene Mutation Database (http://www.hgmd.cf.ac.uk/ac/index.php). HGVS, Human Genome Variation Society (http://www.hgvs.org/). PMID, PubMed paper ID (https://pubmed.ncbi.nlm.nih.gov/).*

In the present study, a hereditary variant, which was designated as *LRP6*: c.2570G > A (p.R857H), was detected in six members of the affected family, including two children in the deciduous phase of tooth development. The four adult patients exhibited remarkable intrafamilial phenotypic variation in the number of missing teeth and the presence of other ectodermal dysplasia-like phenotypes. Yu et al. (2020) reported that this phenotype difference exists between affected individuals; yet, in patients from one family, this is the first time we observed this phenotypic variation. We suppose that this phenotypic difference may be related to the individual genetic background or the non-penetrance of this variant, yet both expanded sample size of patients and specific mechanism has to be studied to clearly elucidate this. Greater attention should be paid to the two children who have not yet developed TA during their future development. On the basis of the common standards for the interpretation of genetic variations (Richards et al., 2015), the novel variant identified in the present study was interpreted as “likely pathogenic,” as evidenced by “PM2, PP1, PP2, PP3, and PP4.” Further functional studies, such as the dimerization of LRP6 and the interaction between LRP6 and Wnts, need to be implemented to demonstrate whether the variant meets the PS3 evidence.

The low-density lipoprotein (LDL)-related receptors (LRPs), such as LRP4, LRP5, and LRP6, represent a group of evolutionary-conserved receptors that are involved in the regulation of a wide range of cellular processes through the modulation of several pathways, including the canonical WNT signaling pathway (Huybrechts et al., 2020). The extracellular region of the majority of the LRP receptors contains one ligand-binding domain comprising cysteine-rich ligand-binding-type repeats and one epidermal growth factor (EGF)-precursor homology domain comprising a YWTD/β-propeller (P) domain and EGF repeats (E) (Huybrechts et al., 2020). Presumably, different types of *LRP6* variations lead to increased or decreased WNT signaling activity, thereby inducing different phenotypes (Huybrechts et al., 2020). Previously reported TA-related variations in the coding regions of *LRP6* were more concentrated in the extracellular PE functional domains (Figure 2C), which could affect the binding of LRP6 to Wnts. In order to determine the effect of the novel variant on protein function, MD predictive simulation was conducted, the results of which indicated that the amino acid residue R857 located in the β-strand formed a hydrogen bond with residue T867. Then, in the mutation-resultant R857H, which replaced the strongly basic arginine with a less basic amino acid, the hydrogen bonds formed by the side chain of R857 residue were expectantly broken, thereby changing its potential distribution. In addition, the effect of this variation on the secondary structure of LRP6 (Figure 3F) could significantly perturb the dimerization of this protein or its binding to Wnt, which is necessary for the formation of the Wnt-Fzd-LRP5-LRP6 complex for the activation of Wnt signaling (Boudin et al., 2013). So far, the evidence suggested the loss of LRP6 protein function, yet it should be demonstrated by further study.

Furthermore, in the *in vitro* study, it was demonstrated that the transcription of *LRP6* was not disrupted by the identified novel variant (Figure 4B). However, according to the WB results, the glycosylated mature form of LRP6 was missing in the Mut samples as only a low MW band appeared (“LRP6” line in Figure 4A; Massink et al., 2015). In this case, the treatment of the cells with Wnt3a could not mediate the proper LRP6 phosphorylation (“p-LRP6” line in Figure 4A; Pan et al., 2008). These results strongly suggested that the p.R857H variant caused loss of function in LRP6. However, the underlying reason for the heterogeneity in the disease phenotype with the same variant and the effect of this variant on the Wnt signaling cascade remain to be explored.

One of the limitations of our study is that there is no *in vivo* validation of the variant for the time being, which will be complemented by further studies. Moreover, limited by funding, we have not tested the novel variant with sufficient interaction experiments between Wnts and LRP6, which will be fully supplemented when we are funded.

## Conclusion

In summary, the present study investigated a large family with TA and identified a novel diagnostic variant in the *LRP6* gene, thereby expanding the mutation spectrum of human tooth agenesis. Moreover, it was confirmed through *in vitro* experiments and intramolecular effects that the identified variant could lead to loss of function in the LRP6 protein, which provided a novel perspective for the verification of the impact of missense variations.

## Data Availability Statement

The datasets presented in this study can be found in online repositories. The names of the repository/repositories and accession number(s) can be found below: https://figshare.com/articles/online_resource/LRP6_zip/14595420, 1.

## Ethics Statement

The studies involving human participants were reviewed and approved by the Ethics Committee of the Capital Medical University Affiliated Beijing Stomatological Hospital. Written informed consent to participate in this study was provided by the participants’ legal guardian/next of kin. Written informed consent was obtained from the individual(s), and minor(s)’ legal guardian/next of kin, for the publication of any potentially identifiable images or data included in this article.

## Author Contributions

D-LZ and KY planned and designed the whole study. Y-XH, C-YG, C-YZ, and XC recruited the family, performed the clinical evaluation, and analyzed the data. Y-SY, Y-QS, and KY conducted genetic detections and corresponding data analysis. Y-XH and X-YD performed structural and MD analysis. C-YG, C-YZ, and KY performed *in vitro* experimental study. All authors wrote, reviewed, and corrected the manuscript.

## Conflict of Interest

The authors declare that the research was conducted in the absence of any commercial or financial relationships that could be construed as a potential conflict of interest.

## Footnotes

1 http://www.omim.org/

2 https://www.illumina.com/science/technology/next-generation-sequencing/plan-experiments/quality-scores.html

3 https://software.broadinstitute.org/gatk/

4 https://www.internationalgenome.org

5 http://exac.broadinstitute.org

6 http://gnomad.broadinstitute.org

7 http://www.megasoftware.net/previousVersions.php

8 http://www1.rcsb.org/
