# Supplementary material for: Investigation of a Novel LRP6 Variant Causing Autosomal-Dominant Tooth Agenesis
Source: Front Genet. 2021 Jul 7;12:688241. doi: 10.3389/fgene.2021.688241 (PMC8292820; doi:10.3389/fgene.2021.688241)

Supplementary Materials

1. Variation filtering criteria:

(1) The variants with AF < 5% defined as the rare SNPs and InDels were kept. And the "P" and "LP" level variants in HGMD & ClinVar were kept.

(2) SNPs were annotated as the conserved nucleotides by requiring scaled CADD scores ⩾ 15; and were predicted to be damaging by prediction algorithm. The criteria we used for the prediction algorithms are: REVEL score > 0.75. Indel variants annotated as loss-of-function variants (frameshift variants, splicing variants, stop-gain, and stop-loss variants) were kept.

(3) After filtering variants with frequency and conservation criteria, XX genes with XX variants were kept and then used to perform Disease annotation.

2. Primer sequences used for Sanger sequencing and Real-time Fluorescence Quantitative PCR

Primer Name	Sequence	Size of Amplicon	Reagent	Condition
LRP6-2570-YDX-F	AGGGCTCAACCGTGAAGTT	471 bp	MIX	Touch down
LRP6-2570-YDX-R	TGCATTCCCCTACCCTTTAACC		MIX	Touch down

3. Protocols for PCR amplification and Real-time Fluorescence Quantitative PCR

(1) PCR amplification condition

Experiment system	PCR conditions	PCR enzyme and buffer
Touch down	Touch-down protocol:<br>The initial step at 95 ℃, 5 min; followed by 21 cycles: 95 ℃, 30 sec→70°C, 30 sec, with decreasing 1°C per cycle→72°C, 30 sec;	TAKARA PrimeSTAR® HS DNA Polymerase (with 0.5M Betaine)

	followed by 25 cycles: 95 ℃, 30 sec→50°C, 30 sec→72°C, 30 sec; final extension at 72 ℃, 10 min.	

(2) Fluorescence Quantitative PCR method and condition

At 48 h after transfection, cells were harvested and total RNA was extracted with an RNeasy Mini Kit (QIAGEN). Reverse transcription was performed with the Prime Script RT Reagent Kit with the gDNA Eraser (Takara).

The expression level was assessed by Fluorescence Quantitative PCR using SYBR Premis Ex Taq II(Perfect Real Time) (Takara) with ABI 7500 system. Data are presented as mean ± standard deviation of three independent real-time PCR experiments. The PCR cycle was as follows: 10 min 95° C, 1 cycle; 10 s 95° C, 30 s 60° C＋fluorescence acquisition, 55 cycles. Values for each gene were normalized to expression level of beta-actin gene (ACTB) via the 2-ΔΔCT method.

Primers for internal control:

ACTB-F	GGCATGGGTCAGAAGGATT
ACTB-R	TGGTGCCAGATTTTCTCCA

4. Results of functional prediction of the missense mutation

Mutation taster：disease causing；polyphen2：PROBABLY DAMAGING with a score of 0.987 (sensitivity: 0.73; specificity: 0.96)；PROVEAN：Variant PROVEAN score Prediction (cutoff= -2.5) R857H -4.042 Deleterious (see images below)

Mutation Taster

mutation t@sting

Prediction	disease causing	Model: *simple_aae*, prob: 0.999999999984837
Summary	• amino acid sequence changed<br>• protein features (might be) affected<br>• splice site changes	hyperlink

analysed issue	analysis result
name of alteration	no title
alteration (phys. location)	chr12:12311984C>T show variant in all transcripts IGV
HGNC symbol	LRP6
Ensembl transcript ID	ENST00000261349
Genbank transcript ID	NM_002336
UniProt peptide	O75581
alteration type	single base exchange
alteration region	CDS
DNA changes	c.2570G>A<br>cDNA.2647G>A<br>g.107963G>A
AA changes	R857H Score: 29 explain score(s)
position(s) of altered AA<br>if AA alteration in CDS	857
frameshift	no

JCVI J. CRAIG VENTER INSTITUTE®

PROVEAN

JCVI H

→ PROVEAN Tools
- PROVEAN Protein
- PROVEAN Protein Batch
  - Human
  - Mouse
- PROVEAN Genome Variants
  - Human
  - Mouse

→ About

→ FAQ

→ News

→ Download

→ Help

→ Contact Us

→ Related Links

PROVEAN Result (Download)

PROVEAN Prediction - Job ID: 1164857894393673

- **Query sequence** (fasta)
- **Supporting sequence set used for prediction**
  Number of sequences: **199** (fasta, E-values)
  Number of clusters: **30**
- **Score thresholds for prediction**
  (1) Default threshold is -2.5, that is:
  -Variants with a score equal to or below -2.5 are considered "deleterious,"
  -Variants with a score above -2.5 are considered "neutral."
  (2) How to use a more stringent threshold.

Variant	PROVEAN score	Prediction (cutoff= -2.5)
R857H	-4.042	Deleterious

- Submitted at 21:22:22 EDT, Sunday, Mar 21, 2021
- Started at 21:22:24 EDT, Sunday, Mar 21, 2021
- Finished at 21:22:46 EDT, Sunday, Mar 21, 2021

* The results are kept for 48 hours.

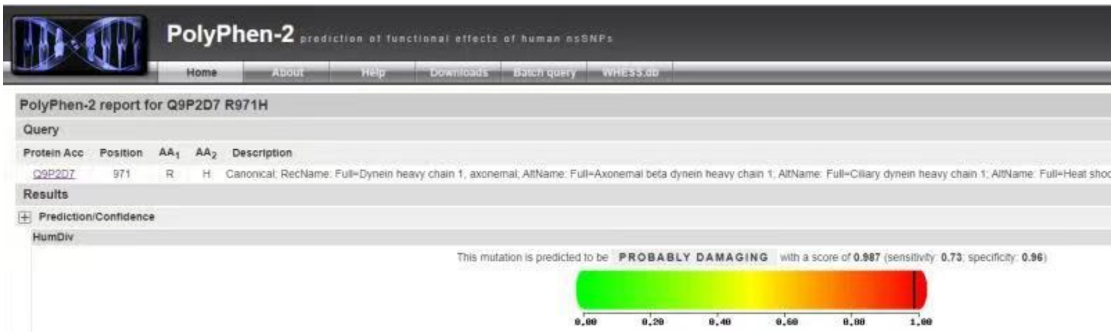

5. Detailed methods and original results of the Western Blot (WB) experiment

(1) WB methods

Cells were washed with cold PBS twice. Then, the total proteins were extracted by RIPA lysis buffer (Beyotime, Jiangsu, China). Lysed cells were centrifugated at 12,000 ×g for 10 min and the protein containing supernatant were collected. The quantification of protein was performed by a BCA assay kit (Abcam, USA). Quantified proteins were then lorded and separated by 10% SDS-PAGE and transferred to PVDF membranes (Millipore Corporation, USA). 5% non-fat milk (BD Bioscience, USA) was applied to block the membrane. Then the membranes were incubated with the primary antibodies against LRP6 (#2560, 1:1000, Cell Signaling Technology), Phospho-LRP6 (#2568, 1:1000, Cell Signaling Technology), GAPDH（60004-1-Ig, 1:20000，proteintech）was incubated overnight at 4 degrees, the membrane was treated with the secondary antibodies (goat anti-rabbit, 1:5000; goat anti-mouse, 1:5000) at room temperature for 2 h. Last, an enhanced chemiluminescence Western blotting system (Pierce, Biotechnology Inc., Rockford, USA) was utilized for quantification.

(2) Original results (raw bands)

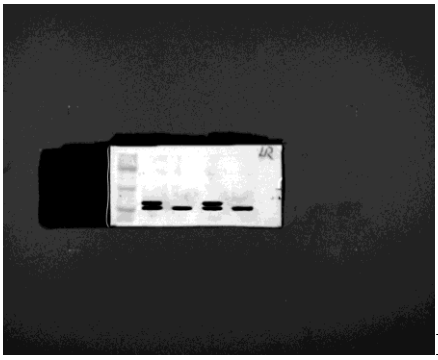
LRP6 (MW, KDa: 180; 210)

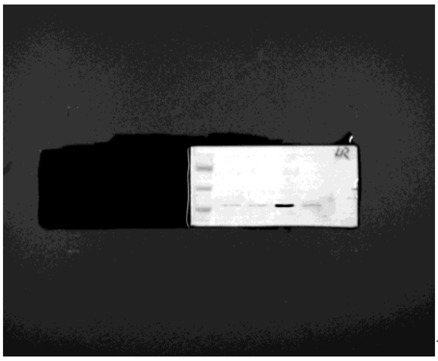
p-LRP6 (MW, KDa: 210)

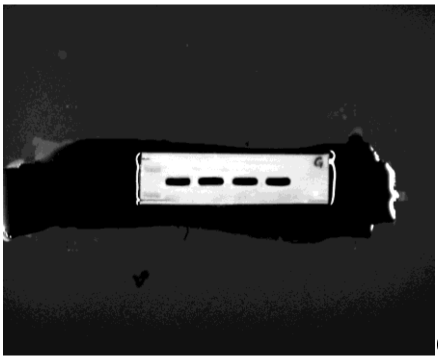
GAPDH (MW, KDa: 36)

6. Stomatological images of the 4 patients in this study.

II-2

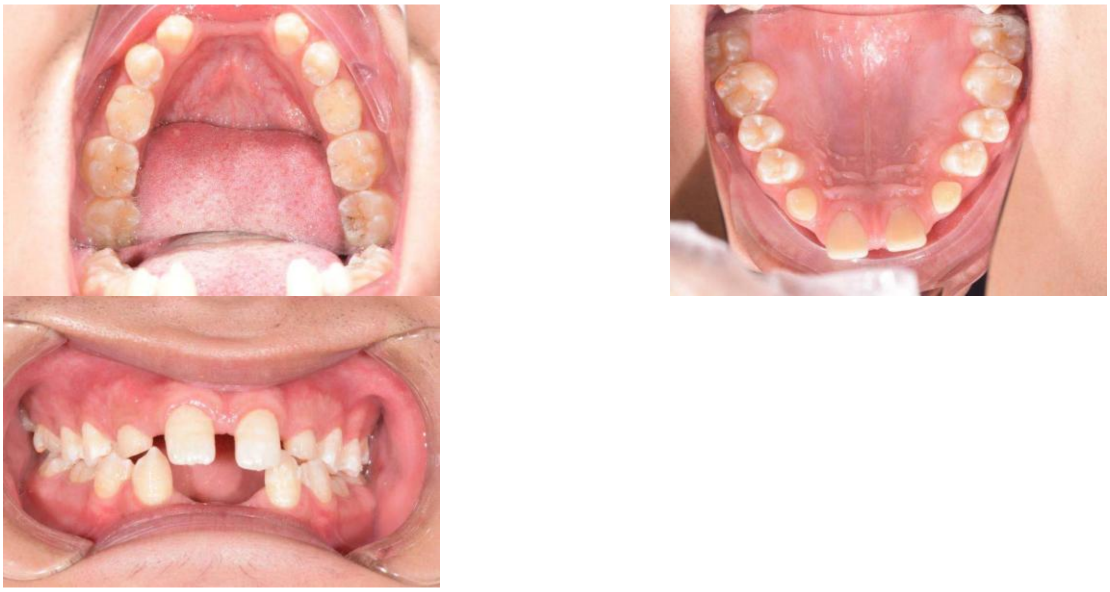

II-3

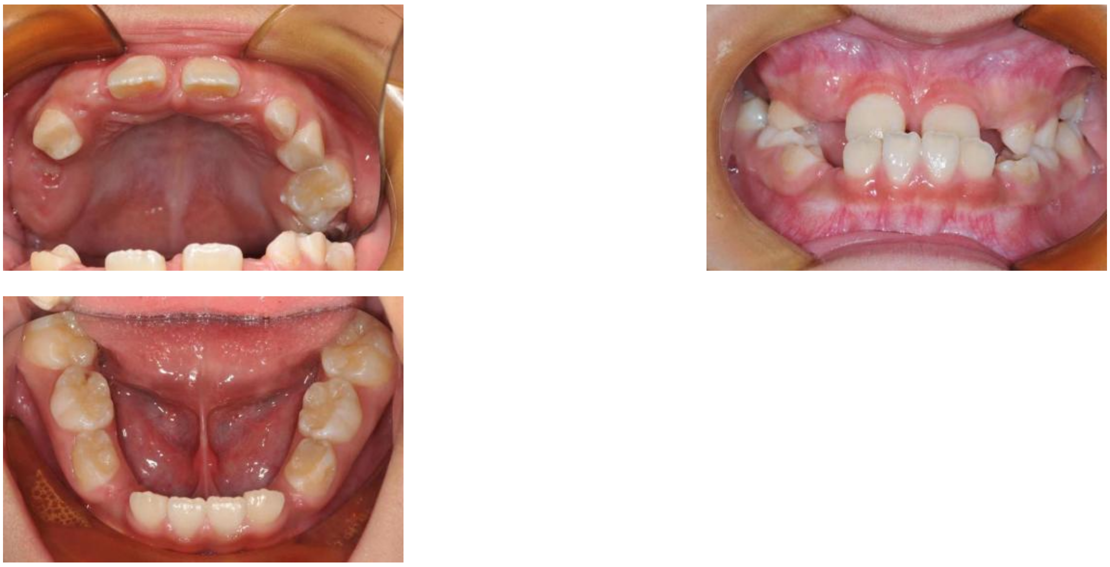

III-2

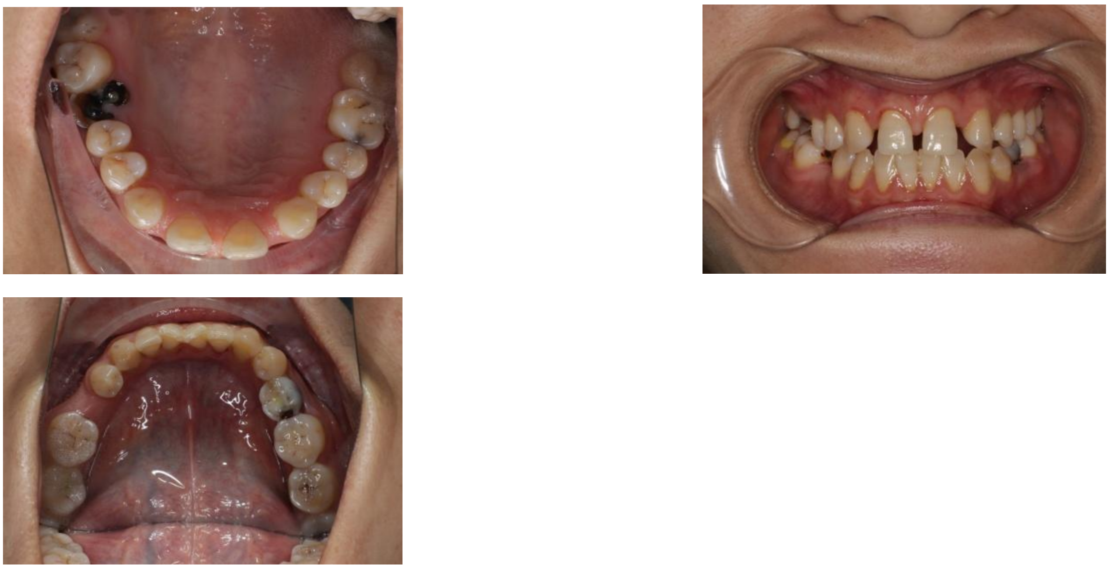

III-3

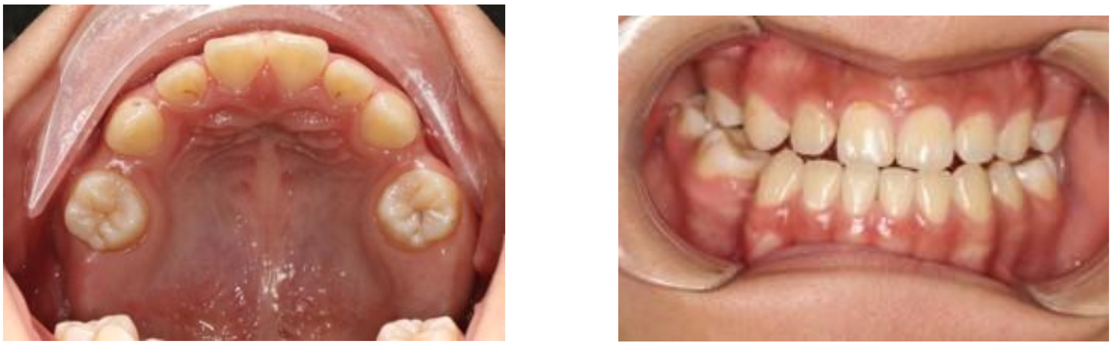

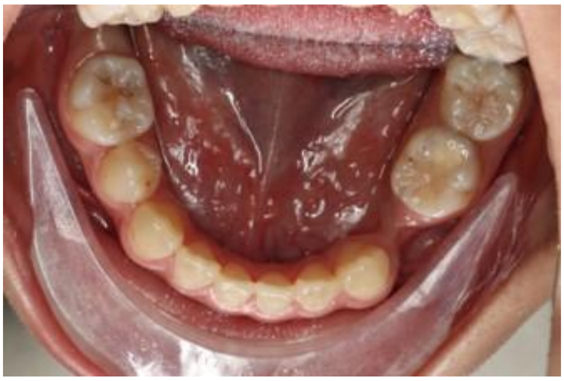

Supplement: Supplementary file 1 [file Data_Sheet_1.PDF]
